# High-density lipopoprotein antioxidant capacity, subpopulation distribution and paraoxonase-1 activity in patients with systemic lupus erythematosus

**DOI:** 10.1186/s12944-016-0229-0

**Published:** 2016-03-22

**Authors:** Krisztina Gaál, Tünde Tarr, Hajnalka Lőrincz, Viktor Borbás, Ildikó Seres, Mariann Harangi, Péter Fülöp, György Paragh

**Affiliations:** Department of Medicine, Faculty of Medicine, University of Debrecen, krt. 98., Debrecen, H-4032 Hungary

**Keywords:** HDL antioxidant capacity, paraoxonase-1, HDL subfractions, SLE, oxidized LDL, cardiovascular disease

## Abstract

**Background:**

The causes of increased cardiovascular risk in systemic lupus erythematosus (SLE) are not understood thoroughly, although presence of traditional cardiovascular risk factors and disease-specific agents were also proposed. In this study, we investigated the quantitative changes in the lipid profile, as well as qualitative characteristics of high-density lipoprotein (HDL) and markers of inflammation and disease activity in SLE patients.

**Methods:**

Lipoprotein levels were determined in 51 SLE patients and 49 healthy controls, matched in age and gender. HDL antioxidant capacity was determined spectrophotometrically with a cell-free method of hemin-induced low-density lipoprotein (LDL) oxidation. Polyacrylamide gel-electrophoresis was used for HDL subfraction analysis. Human paraoxonase-1 (PON1) activity, apolipoprotein A1 (ApoA1) and oxidized LDL concentrations, as well as interleukin-6, high-sensitivity C-reactive protein, serum amyloid A and monocyte chemotactic protein-1 levels were determined.

**Results:**

HDL-cholesterol and ApoA1 concentrations decreased significantly in SLE subjects. Also, PON1 arylesterase activity (125.65 ± 26.87 vs. 148.35 ± 39.34 U/L, *p = 0.001*) and total HDL antioxidant capacity (165.82 ± 58.28 % vs. 217.71 ± 54.36 %, *p < 0.001*) were significantly reduced in patients compared to controls. Additionally, all HDL subfraction concentrations were significantly decreased in patients, while the levels of the examined inflammatory markers were significantly elevated in SLE subjects. The latter correlated positively with disease activity, and negatively with HDL concentration and total HDL antioxidant capacity, respectively. PON1 arylesterase activity and erythrocyte sedimentation rate were independent predictors of total HDL antioxidant capacity.

**Conclusions:**

Induced by the systemic inflammation, altered composition and antioxidant activity may diminish the anti-atherogenic effect of HDL and therefore may contribute to the increased cardiovascular risk of SLE patients.

## Background

Several investigations attempted to clarify the background of increased cardiovascular (CV) morbidity and mortality in systemic lupus erythematosus (SLE), which is one of the most common systemic autoimmune diseases [1]. SLE patients suffer from accelerated atherosclerosis, featured by increased carotid intima-media thickness (cIMT), carotid plaques and endothelial dysfunction, as well as impaired flow-mediated dilation (FMD) in the brachial artery [2]. Higher prevalence of traditional cardiovascular risk factors and disease specific agents were also suggested to play a role in the course of plaque formation.

Previous studies reported the “lupus pattern” of lipoproteins in SLE that is characterized by elevated triglyceride (TG) and very-low-density lipoprotein (VLDL) concentrations together with decreased high-density lipoprotein cholesterol (HDL) levels, usually occurring in the active phases of the disease [3, 4]. Others reported increased low-density lipoprotein (LDL) and total cholesterol (TC) concentrations during the periods of lower disease activity [3, 5].

Inflammation is a key factor both in SLE and atherosclerosis. During acute or chronic inflammation, HDL suffers substantial changes both in composition and function, diminishing its anti-atherogenic effects. The concentration of apolipoprotein A1 (ApoA1), which is the main structural protein of HDL, decreases during the acute phase response. Also, myeloperoxidase-dependent chlorination of ApoA1 side-chains impairs the ability of HDL to facilitate reverse cholesterol transport (RCT) via the ATP-binding cassette transporter A1 (ABCA1) pathway [6]. Previous data suggest that the direct anti-oxidant effect of HDL on LDL oxidation, measured as a reduction in lipid peroxides, is likely mediated by HDL-linked paraoxonase-1 (PON1) [7, 8]. This major antioxidant effect might be mitigated in inflammatory conditions. Qualitative changes of the HDL reported in inflammatory states are partly the direct results of altered gene expression of HDL-associated proteins including serum amyloid A that is modulated by inflammatory cytokines. Furthermore, altered lipoprotein metabolism may indirectly influence the composition and function of HDL. Both of these mechanisms may be responsible for the decrease in the ApoA1 levels. Reverse cholesterol transport is also impaired during acute phase reaction [9].

Indeed, diminished PON1 activity was reported in SLE patients compared to healthy controls [10]. Incorporation of serum amyloid A (SAA) into the HDL particle results in a structural modification of the lipoprotein with a consequent functional deficiency, characterized by reduced inhibition of monocyte chemoattractant protein-1 (MCP-1) production in vascular smooth muscle cells [11]. High levels of acute phase reactant C-reactive protein (CRP), SAA and increased erythrocyte sedimentation rate (ESR) were described previously in SLE patients together with elevated MCP-1 and interleukin-6 (IL-6) concentrations, which showed positive associations with coronary calcification [12, 13].

By now, just a few studies analyzed the distribution of the HDL subfractions in SLE and the results are controversial. Regardless their history of cardiovascular disease, small HDL subfractions (sHDL) were found to be less prevalent in SLE patients than in healthy controls [14]. Moreover, in a cohort of 69 SLE patients, sHDL particles were in direct correlation with cIMT, C3, C4 and CH50 complement levels [15].

A cell-free method was developed by Navab et al. to determine the inhibitory effect of HDL on LDL oxidation [16]. Proinflammatory HDL (piHDL) was detected if the fluorescent signal generated upon oxidation turned out to be larger in the joint presence of HDL and LDL, than LDL alone. It was reported that almost half of the SLE patients have piHDL (44.7 % vs. 4.1 % in healthy controls), with increasing prevalence further up to 86.7 % in those with concurrent carotid plaque [17, 18]. In SLE patients, the prevalence of piHDL correlated with the occurrence of carotid plaque and cardiovascular complications and also with the concentrations of oxidized LDL (oxLDL) [17, 18].

Using hemin as a physiological pro-oxidant, we also developed a cell-free method to assess the antioxidant capacity of HDL [19]. Data were suitable for multiplication with HDL concentration, resulting total HDL antioxidant capacity, which is able to characterize antioxidant attribute and concentration of HDL simultaneously.

This study of SLE patients and healthy controls was organized to compare HDL antioxidant capacities by the cell-free method of hemin-induced LDL oxidation and to compare lipoprotein and ApoA1 concentrations, PON1 activities and HDL subfractions. To get a more complex picture on the relationship between inflammation and lipids in SLE, associations between inflammatory markers and lipid parameters were also examined.

## Methods

### Study population

51 patients with SLE and 49 healthy controls were enrolled. Patients were treated in the Division of Clinical Immunology, Department of Medicine, University of Debrecen. All patients fulfilled at least four of the revised American College of Rheumatology (ACR) classification criteria for SLE [20]. Controls were recruited from health care professionals and healthy local habitants. Subjects with renal failure (GFR < 60 ml/min/1,73 m^2^), diabetes mellitus or statin use were excluded due to their impact on HDL anti-inflammatory function [21, 22]. Patients and controls were matched in age, gender and smoking habits, for those affect lipid profile and HDL antioxidant enzyme activities [23, 24]. The study was approved by the Ethical Committee of University of Debrecen and all participants gave written informed consent.

Clinical data on age, gender, smoking habits, statin intake, hypertension (use of antihypertensive medication or blood pressure ≥ 140/90 mmHg), diabetes mellitus (use of antidiabetic medication or fasting blood glucose ≥ 7 mmol/L or postprandial blood glucose ≥11.1 mmol/L), previous cerebrovascular or cardiovascular disease (transient ischemic attack, ischemic or hemorrhagic stroke, myocardial infarct or angina pectoris) were collected in both study groups. Disease activity was assessed by the SLEDAI score [25] and disease duration, disease manifestations as well as current therapy were also recorded.

Among the 51 patients, 42 used methylprednisolon, 20 azathioprine, 11 chloroquine and 2 cyclosporine A. Average daily steroid dose was 4 mg, ranging from 0 to 32 mg; while 5 patients did not take any drugs at the time of blood sampling.

### Sample collection and laboratory measurements

Venous blood samples were collected after a 12-h fast. Routine laboratory parameters, including blood counts, glucose level, ESR, renal and hepatic function tests were determined from fresh sera with a Cobas c501 analyzer (Roche Ltd, Mannheim, Germany). High-sensitivity C-reactive protein (hsCRP), ApoA1, apolipoprotein B (ApoB) and lipoprotein (a) [Lp(a)] concentrations were assessed by immunoturbidimetric assays. Total cholesterol (TC), triglyceride (TG), high-density lipoprotein cholesterol (HDL-C) and low-density lipoprotein cholesterol (LDL-C) levels were measured by enzymatic colorimetric tests (Modular P-800 Analyzer, Roche/Hitachi). Autoantibodies specific for SLE and antiphospholipid syndrome (a-dsDNA, a-Sm, a-SSA, a-SSB; a-CL IgA, a-CL IgG, a-CL IgM, a-B2 IgA, a-B2 IgG, a-B2 IgM), immunoglobulin (IgA, IgG, IgM), complement (C3, C4) and immune complex concentrations were tested with commercially available enzyme-linked immunosorbent assay (ELISA) kits. Cytokines, acute phase markers and oxLDL were tested with commercially available ELISA assays (IL-6: R&D Systems Inc., Minneapolis, USA; serum amyloid A (SAA): Antigenix America Inc., NY, US; MCP-1: R&D Systems Inc., Minneapolis, USA; oxLDL: Biomedica, Vienna, Austria). Assays were performed according to the recommendations of the manufacturers.

### Determination of HDL antioxidant capacity

Our method to determine HDL antioxidant capacity has been described previously [19]. The effect of HDL was measured by heme-catalyzed oxidation of LDL in the absence and presence of HDL. Using HDL samples of identical cholesterol concentrations, the inhibitory effect of HDL on LDL oxidation (termed specific HDL antioxidant capacity) was assessed by measuring ΔT_Vmax_ of hemin-catalyzed LDL oxidation in the absence and presence of HDL. HDL was isolated from sera with density gradient ultracentrifugation and hydrogen peroxide was used to split the heme ring and release redox-active iron in order to accelerate the reaction. LDL oxidation was followed spectrophotometrically by measuring hemin degradation. The HDL antioxidant capacity was expressed as the percentage ratio of ΔT_Vmax_ in the presence and absence of HDL [19, 26].

Total HDL antioxidant capacity values were calculated by multiplying the specific HDL antioxidant capacity values with the HDL concentrations of the study participants.

### Determination of human paraoxonase-1 enzyme activities and phenotype distribution

PON1 paraoxonase activity was analyzed on a microtiter plate by a kinetic, semiautomated method using paraoxon (O,O-diethyl-O-p-nitrophenyl-phosphate, Sigma Aldrich, Hungary) as a substrate. PON1 arylesterase activity was assayed with a phenylacetate substrate (Sigma Aldrich, Hungary) and the hydrolysis of phenylacetate was monitored at 270 nm [10] The dual substrate method was used to determine the phenotypic distribution of PON1, (ratio of salt-stimulated paraoxonase to the hydrolysis of phenyl acetate): ratio < 3.0 for AA, ratio between 3.0 and 7 for AB and ratio >7.0 for BB phenotype.

### HDL subfraction analysis

HDL subfractions were detected by an electrophoretic method on polyacrylamide gel with the Lipoprint System (Quantimetrix Corp., CA, USA) according to manufacturer’s instructions. Lipoprint separates HDL subfractions from human serum on the basis of their size applying preloaded gel tubes for HDL determinations. Samples from 51 SLE patients and from 40 controls were analyzed.

Concisely, 25 μl serum was added to the polyacrylamide gel tubes along with 300 μl loading gel solution. The tubes contained Sudan Black as a lipophilic dye and were photopolimerized at room temperature for 30 min. Electrophoresis with tubes containing sera samples or the manufacturer’s quality controls was performed at a constant of 3 mA/tube for 50 min. Subfraction bands were scanned with an ArtixScan M1 digital scanner (Microtek International Inc., CA, USA) and were identified by their mobility (Rf) using VLDL as the starting (Rf 0°0) and albumin as the ending (Rf 1°0) reference point.

Ten HDL subfractions were differentiated between LDL and albumin peaks, and were grouped into three major classes: large (from HDL1 to HDL3), intermediate (from HDL4 to HDL7) and small (HDL8 to HDL10) HDL subfractions. Cholesterol concentrations of the HDL particle subsets were calculated by multiplying the total cholesterol concentration of the samples by the relative area under the curve (AUC) of the subfraction bands.

### Statistical analysis

The normality of continuous variables was assessed by the Kolmogorov-Smirnov test. Normally distributed data were expressed as mean (±SD), while data not normally distributed were expressed as median (interquartile range [IQR]). Continuous variables were compared by Student’s *t*-test or Mann–Whitney *U* test, as appropriate. Associations between continuous variables were assessed by Pearson’s or Spearman’s correlation coefficient, depending on the normality of data. Multiple regression analysis (backward-stepwise method) was performed to determine variables best predicted the total HDL antioxidant activity. The level of significance was set at *P* < 0.05. Analyses were performed on Statistica – Version 8 computer software (StatSoft Inc.).

## Results

### Basic characteristics and lipid profile

Basic clinical and laboratory characteristics including lipoprotein concentrations of the study groups are presented in Table 1. SLE patients were relatively young with a mean disease duration of 6.59 ± 5.26 years and their SLEDAI score was 4.0 [2.0 – 6.0], ranging from 0 to 38. Although the glucose level was significantly higher in SLE patients compared to controls, it was still in the normal range. Significantly lower HDL and ApoA1 levels, as well as significantly higher triglyceride and ApoB100 levels were found in SLE patients compared to controls.

**Table 1:** Basic clinical and laboratory characteristics of the study groups

Characteristics	SLE patients (*n* = 51)	Controls (*n* = 49)	*P* Value
Gender (female/male)	44/7	41/8	0.717
Age (yr)	31.82 ± 6.40	31.8 ± 6.81	0.983
BMI (kg/m^2^)	24.45 ± 4.13	23.86 ± 4.28	0.489
Current smoker (%)	31.37	26.53	0.484
Systolic blood pressure (mmHg)	115 (110–120)	117 (110–120)	0.952
Diastolic blood pressure (mmHg)	70 (70–80)	78 (70–80)	0.592
Glucose (mmol/L)	**4.89 ± 0.77**	4.32 ± 0.57	**<0.001**
Total cholesterol (mmol/L)	4.57 ± 1.12	4.81 ± 0.80	0.222
LDL-cholesterol (mmol/L)	2.60 ± 0.84	2.71 ± 0.69	0.473
HDL-cholesterol (mmol/L)	1.26 ± 0.47	**1.71 ± 0.45**	**<0.001**
Non-HDL cholesterol (mmol/L)	3.22 ± 1.23	3.10 ± 0.78	0.573
Triglycerides (mmol/L)	**1.25 (0.9-1.9)**	0.90 (0.7-1.4)	**0.005**
Apolipoprotein A1 (g/l)	1.39 ± 0.35	**1.66 ± 0.41**	**<0.001**
Apolipoprotein B-100 (g/l)	**0.86 ± 0.26**	0.76 ± 0.22	**0.047**
Lipoprotein (a) (mg/l)	106 (29–294)	76 (29–169)	0.240
ApoA1/HDL (g/mmol)	**1.17 ± 0.26**	0.99 ± 0.17	**<0.001**

Statistically significant differences are marked in bold

### Antioxidant capacity and PON1 phenotype distribution

Compared to controls, SLE patients displayed non-significantly decreased PON1 paraoxonase and significantly decreased PON1 arylesterase activities (Table 2). To assess whether altered PON1 activity was due to the decreased HDL level found in SLE group, we standardized the enzyme activity for the HDL concentration (PON1/HDL ratio). We found that the standardized enzyme activities were significantly higher in the SLE patients compared to controls. Additionally, significantly lower (−23.83 %) total HDL antioxidant, while slightly, but significantly higher (3.72 %) specific HDL antioxidant capacities were found in the SLE patients.

**Table 2:** HDL antioxidant enzyme activities and inflammatory markers

Characteristics	SLE patients (*n* = 51)	Controls (*n* = 49)	*P* Value
PON1 paraoxonase activity (U/l)	118.01 ± 85.49	124.68 ± 102.96	0.857
PON1 arylesterase activity (U/l)	125.65 ± 26.87	**148.35 ± 39.34**	**0.001**
Paraoxonase activity/HDL	**98.48 ± 69.92**	76.20 ± 64.34	**0.025**
Arylesterase activity/HDL	**109.05 ± 38.23**	93.51 ± 33.30	**0.036**
Total HDL antioxidant capacity (%)	165.82 ± 58.28	**217.71 ± 54.36**	**<0.001**
OxLDL (mg/l)	**3.55 ± 4.19**	2.63 ± 3.22	0.291
HsCRP (mg/l)	**5.02 ± 6.64**	1.68 ± 1.88	**0.002**
ESR (mm/hour)	**24.38 ± 17.96**	7.90 ± 5.93	**<0.001**
IL-6 (ng/l)	**0.54 (0–3.25)**	0 (0–0.17)	**0.002**
SAA (ug/l)	**19017.07 ± 25899.25**	2666.05 ± 2301.11	**<0.001**
MCP-1 (ng/l)	**473.24 (342.72-599.16)**	309.72 (242.91-353.86)	**<0.001**
SAA/HDL (ug/mmol)	**18217.14 ± 29453.17**	1692.24 ± 1459.34	**<0.001**

*ESR* erythrocyte sedimentation rate; *HDL* high-density lipoprotein; *hsCRP* high-sensitivity C-reactive protein; *IL-6* interleukin-6; *MCP-1* monocyte chemoattractant protein-1; *ox-LDL* oxidized low-density lipoprotein; *PON1* paraoxonase-1; *SAA* serum amyloid A

Statistically significant differences are marked in bold

The PON1 AA phenotype, indicating the lowest PON1 enzyme activity was present in 94 % of the SLE patients and 87.23 % of controls. AB phenotype with intermediate enzyme activity was found in 6 % of the patients vs. 12.77 % of the controls. BB phenotype, the indicator of the highest PON1 enzyme activity, was not present in either of the study groups. Allele B of *PON1* associated with higher enzyme activity was twice as frequent in healthy subjects vs. SLE patients (6.38 % vs. 3 %).

Inflammatory markers including hsCRP levels and ESR were significantly increased in the SLE patients. Also, concentrations of IL-6, MCP-1 and SAA were found to be significantly increased in the patient group.

### HDL subfraction analysis

Compared to the control individuals, Lipoprint analysis of the sera showed uniformly and significantly lower amounts of the HDL fractions in the SLE patients (Table 3). However, there were no significant differences in the distribution of the HDL subfractions between the two groups.

**Table 3:** HDL subfraction analysis

Subclasses	SLE patients (*n* = 51)	Controls (*n* = 49)	*P* Value
Large HDL (mg/dl)^a^	18.37 ± 9.91	**23.88 ± 10.33**	**0.012**
Intermediate HDL (mg/dl)^b^	22.98 ± 6.48	**32.15 ± 6.77**	**<0.001**
Small HDL (mg/dl)^c^	6.51 ± 2.47	**10.08 ± 3.53**	**<0.001**
Small HDL-Large HDL ratio	0.41 ± 0.19	0.53 ± 0.41	0.077
Large HDL (%)^a^	36.57 ± 7.27	34.79 ± 8.02	0.268
Intermediate HDL (%)^b^	49.32 ± 5.42	49.50 ± 4.78	0.869
Small HDL (%)^c^	14.08 ± 3.94	15.69 ± 5.63	0.114

^a^sum of HDL1, HDL2 and HDL3

^b^sum of HDL4, HDL5, HDL6 and HDL7

^c^sum of HDL8, HDL9, and HDL10

Statistically significant differences are marked in bold

### Correlations between markers of oxidative stress and inflammation

**Fig. 1:**
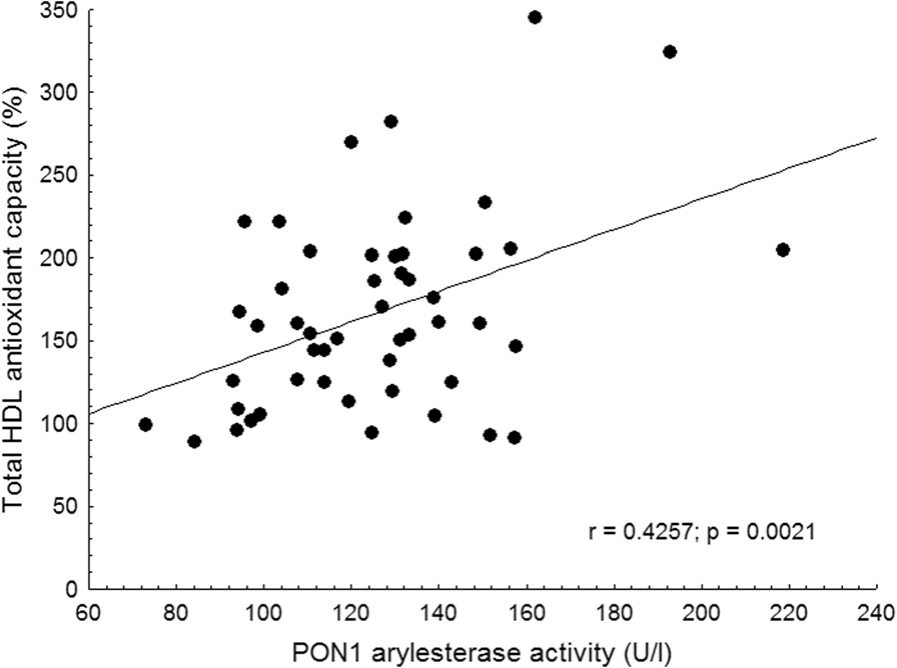
Correlation of total HDL antioxidant capacity and PON1 arylesterase activity in the SLE group

**Fig. 2:**
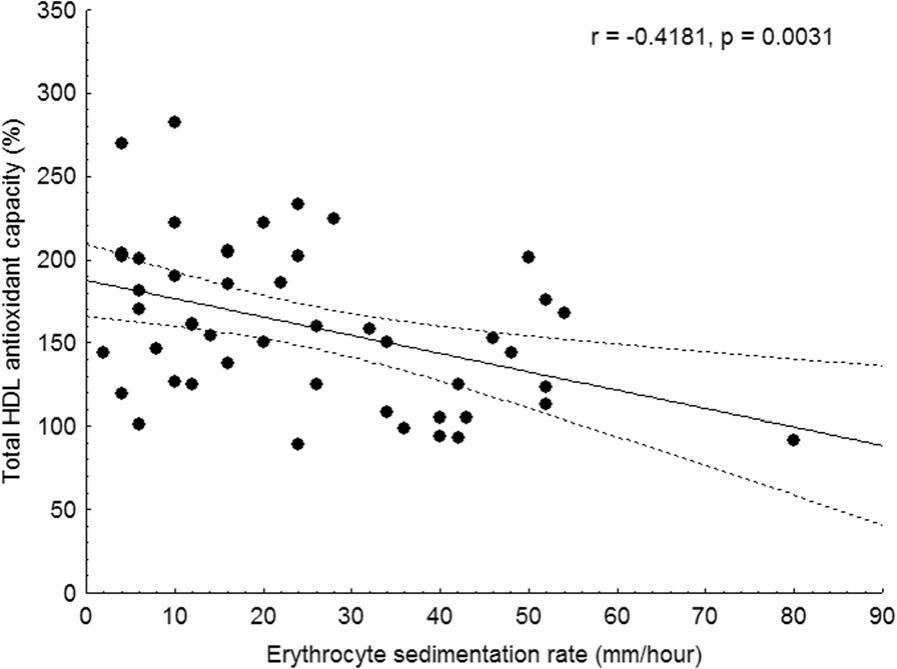
Correlation of total HDL antioxidant capacity and ESR in SLE patients

**Fig. 3:**
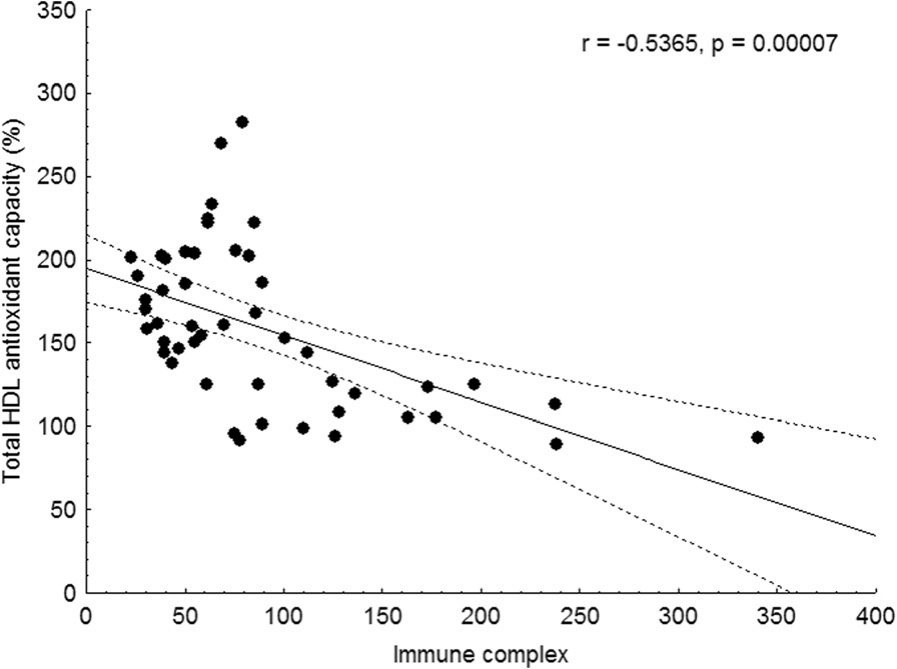
Correlation of total HDL antioxidant capacity and immune complex concentration in SLE

Both HDL-C and total HDL antioxidant capacity correlated positively with PON1 arylesterase (Fig. 1) and paraoxonase activity in SLE patients, but not in controls. HDL-C and total HDL antioxidant capacity showed significant negative correlations with the inflammatory markers including ESR (Fig. 2), hsCRP, IL-6, MCP-1, and SAA (Table 4). HDL antioxidant capacity showed a significant inverse relationship with immune complex level (Fig. 3); however, we could not find significant correlations between disease activity index and ApoA1, HDL-C or total HDL antioxidant capacity *(data not shown)*.

**Table 4:** Correlations between markers of oxidative stress and inflammation in SLE patients

	HDL-C		total HDL antioxidant capacity	
	r	P	r	*P*
PON1 arylesterase activity	0.378	<0.01	0.425	<0.01
PON1 paraoxonase activity	0.289	<0.05	0.290	<0.05
ESR	−0.489	<0.0001	−0.419	<0.01
hsCRP	−0.314	<0.01	−0.347	<0.001
IL-6	−0.489	<0.0001	−0.500	<0.0001
MCP-1	−0.467	<0.0001	−0.413	<0.0001
SAA	−0.288	<0.01	−0.283	<0.01

*ESR* erythrocyte sedimentation rate; *HDL* high-density lipoprotein; *hsCRP* high-sensitivity C-reactive protein; *IL-6* interleukin-6; *MCP-1* monocyte chemoattractant protein-1; *PON1* paraoxonase-1; *SAA* serum amyloid A

In the SLE patients, PON1 paraoxonase activity correlated positively with the proportion of large HDL % (*r* = 0.34808, *P* = 0.0123) and with its concentration (*r* = 0.33598, *P* = 0.0159), while it showed a negative association with the proportion of intermediate HDL (*r* = −0.38145, *P* = 0.0057). In the controls, PON1 paraoxonase and arylesterase activities correlated only with the concentration of the intermediate HDL *(data not shown).* PON1 arylesterase activity showed positive associations with the concentrations of all HDL subpopulations, but it was most pronounced with small HDL (*r* = 0.40204, *P* = 0.0038) in the SLE group. Also, anti-SSB showed an inverse relationship with PON1 arylesterase activity (*r* = −0.33061, *P* = 0.019).

Disease activity, evaluated by the SLEDAI score, showed a significant positive correlation with oxLDL concentration (*r* = 0.34716, *P* = 0.0126). In the patients, OxLDL levels correlated positively with SAA concentration (*r* = 0.31279, *P* = 0.0344), ESR (*r* = 0.30226, *P* = 0.0368) and hsCRP levels (*r* = 0.27253, *P* = 0.0582) concentrations and oxLDL level was also associated with higher concentration of ApoB (*r* = 0.31239, *P* = 0.0256). Furthermore, oxLDL level was related with decreased large HDL ratio (*r* = −0.27118, *P* = 0.0595) and with increased intermediate HDL ratio (*r* = 0.39379, *P* = 0.0042), respectively; while showing a positive association with fibrinogen concentration (*r* = 0.51245, *P* = 0.0001).

To test whether the associations detected in the univariate analyses were independent of other laboratory parameters, we carried out a multiple regression analysis with total HDL antioxidant activity as the dependent variable. The model included IL-6, hsCRP, MCP-1, SAA, ESR and PON1 arylesterase activity. Total HDL antioxidant activity turned out to be best predicted by the PON1 arylesterase activity (*p* < 0.05, beta = 0.348) and ESR (*p* < 0.01, beta = 0.61).

## Discussion

Characterized by the SLEDAI score, our young SLE cohort had a relatively high disease activity. Therefore, it is not surprising that their basic lipoprotein profile showed the typical “lupus pattern” [3, 4], featured by elevated triglyceride and decreased HDL-C levels without significant changes in total and LDL-C concentrations. The concentration of ApoA1, which is the major apolipoprotein of HDL, was also significantly decreased in patients. Since intact function of ApoA1 is required to the activation of ABCA1-dependent reverse cholesterol transport and also to LCAT and PON1 enzyme activation, decreased concentration or altered structure of ApoA1 may lead to deficient HDL function [6, 27]. Despite the LDL levels being similar in our study groups, ApoB concentration was higher in our lupus patients compared to healthy subjects. Previously, increased ApoB level was found to be associated to larger plaque burden and larger presence of non-calcified plaques in patients with ischemic heart disease [28]. In a previous study on 87 SLE patients with inactive disease, ApoB concentration was shown to be independently associated with pulse wave velocity [29]. Deposition of ApoB within the arterial wall, which is one of the initiating steps of atherosclerosis, was detected in coronary plaques by fluorescent microscopy [30].

Using our method of hemin-induced LDL oxidation, we determined the specific antioxidant activity of HDL in the study groups that both were characterized by unaltered total cholesterol concentrations. HDL protected test LDL from oxidation, lengthening the time period until the LDL oxidation reached its maximum reaction velocity. None of the examined HDLs were found to be “proinflammatory” according to our antioxidant activity results. Surprisingly, specific HDL antioxidant capacity was significantly elevated in patients; while, at the same time, total HDL antioxidant capacity was appreciably decreased in these individuals. Our data might indicate altered RCT of the patients that provides less cholesterol content, thus leading to increased protein/cholesterol ratio in the HDL particles. We suppose that higher enzyme concentration in the reaction mixture results in enhanced antioxidant activity, although not necessarily providing greater antioxidant protection.

Our findings differ from those reported by McMahon et al. [17]. They detected the presence of proinflammatory HDL in 44.7 % of their SLE cohort compared to the 4.1 % observed in the control group. In another study from this research group, the overall frequency of piHDL was 48.2 % among SLE patients; while 86.7 % of the patients with carotid plaque carried piHDL contrary to 40.7 % of those without plaque [18]. The different study populations and methods applied in these studies may explain the conflicting results. Their patients had significantly decreased LDL-C, but similar TC, HDL-C and TG concentrations than controls; while our group of SLE subjects displayed diminished HDL-C and elevated TG levels. Additionally, McMahon et al. used an end-point measurement in contrast to our kinetic reaction and the utilized pro-oxidants were also different. Although piHDL was not detected in our study, total HDL antioxidant activity of the lupus patients were significantly decreased and it turned out to be best predicted by PON1 arylesterase activity and ESR. These findings highlight the role of the PON1 enzyme and the inflammatory processes in the regulation of the HDL antioxidant status in SLE.

In a previous study by Kiss et al., decreased PON1 paraoxonase activity was found in SLE patients, while PON1 arylesterase remained unaltered [10]. Similarly to their results, we could not find significant differences between patients and controls regarding PON1 phenotype distribution; and none of our study participants had the high activity BB phenotype.

HDL maturation is a complex process followed by structural and functional changes that are reflected by the HDL subfraction distribution. The different subsets of the lipoprotein particles carry various enzymes and structural proteins that alter their function. Small dense HDL particles are the main carriers of the antioxidant enzymes including PON1, and they are also important lipid acceptors during RCT. Examining HDL subpopulations in both study groups, they were measured to be equally diminished in the SLE patients and correlated both with decreased HDL antioxidant activity and lower PON1 arylesterase activity.

## Conclusion

In conclusion, total HDL antioxidant capacity was severely diminished in SLE patients compared to controls. The subsequent analyses of HDL subpopulations revealed equally decreased HDL subfractions, while we could not detect the presence of proinflammatory HDL in the SLE subjects. Small HDL concentration and HDL antioxidant capacity showed positive correlations with PON1 arylesterase activity and inverse relationships with inflammatory markers (MCP-1, IL-6, hsCRP), and disease activity (SLEDAI). ESR and PON1 arylesterase activity were independent predictors of total HDL antioxidant activity. Due to the systemic inflammation in SLE, altered composition and antioxidant activity of HDL may diminish its anti-atherogenic effect and therefore may contribute to the increased cardiovascular risk of SLE patients. Negative correlations between HDL antioxidant capacity and activity markers including ESR and immune complex level highlight the role of disease activity in decreased protection against atherogenesis in SLE.

## Abbreviations

Apo A1: apolipoprotein A1
ApoB: apolipiprotein B
cIMT: carotid intima-media thickness
ESR: erythrocyte sedimentation rate
FMD: flow-mediated dilation
HDL: high-density lipoprotein
hsCRP: high-sensitivity C-reactive protein
IL-6: interleukin-6
LDL: low-density lipoprotein
MCP-1: monocyte chemotactic protein-1
PON-1: paraoxonase-1
RCT: reverse cholesterol transport
SAA: serum amyloid A
SLE: systemic lupus erythematosus
SLEDAI: systemic lupus erythematosus disease activity index
TC: total cholesterol
TG: triglyceride
VLDL: very-low-density lipoprotein

## References

[1] Manzi S, Meilahn EN, Rairie JE, Conte CG, Medsger TA, Jansen-McWilliams L, D'Agostino RB, Kuller LH (1997). Age-specific incidence rates of myocardial infarction and angina in women with systemic lupus erythematosus: comparison with the Framingham Study. Am J Epidemiol.

[2] El-Magadmi M, Bodill H, Ahmad Y, Durrington PN, Mackness M, Walker M, Bernstein RM, Bruce IN (2004). Systemic lupus erythematosus: an independent risk factor for endothelial dysfunction in women. Circulation.

[3] Ilowite NT, Samuel P, Ginzler E, Jacobson MS (1988). Dyslipoproteinemia in pediatric systemic lupus erythematosus. Arthritis Rheum.

[4] Borba EF, Bonfá E (1997). Dyslipoproteinemias in systemic lupus erythematosus: influence of disease, activity, and anticardiolipin antibodies. Lupus.

[5] Nuttall SL, Heaton S, Piper MK, Martin U, Gordon C (2003). Cardiovascular risk in systemic lupus erythematosus--evidence of increased oxidative stress and dyslipidaemia. Rheumatology (Oxford).

[6] Zheng L, Settle M, Brubaker G, Schmitt D, Hazen SL, Smith JD, Kinter M (2005). Localization of nitration and chlorination sites on apolipoprotein A-I catalyzed by myeloperoxidase in human atheroma and associated oxidative impairment in ABCA1-dependent cholesterol efflux from macrophages. J Biol Chem.

[7] Mackness MI, Arrol S, Durrington PN (1991). Paraoxonase prevents accumulation of lipoperoxides in low-density lipoprotein. FEBS Lett.

[8] Watson AD, Berliner JA, Hama SY, La Du BN, Faull KF, Fogelman AM, Navab M (1995). Protective effect of high density lipoprotein associated paraoxonase. Inhibition of the biological activity of minimally oxidized low density lipoprotein. J Clin Invest.

[9] Rohrer L, Hersberger M, von Eckardstein A (2004). High density lipoproteins in the intersection of diabetes mellitus, inflammation and cardiovascular disease. Curr Opin Lipidol.

[10] Kiss E, Seres I, Tarr T, Kocsis Z, Szegedi G, Paragh G (2007). Reduced paraoxonase1 activity is a risk for atherosclerosis in patients with systemic lupus erythematosus. Ann N Y Acad Sci.

[11] Tölle M, Huang T, Schuchardt M, Jankowski V, Prüfer N, Jankowski J, Tietge UJ, Zidek W, van der Giet M (2012). High-density lipoprotein loses its anti-inflammatory capacity by accumulation of pro-inflammatory-serum amyloid A. Cardiovasc Res.

[12] Asanuma Y, Chung CP, Oeser A, Shintani A, Stanley E, Raggi P, Stein CM (2006). Increased concentration of proatherogenic inflammatory cytokines in systemic lupus erythematosus: relationship to cardiovascular risk factors. J Rheumatol.

[13] Rho YH, Chung CP, Oeser A, Solus J, Raggi P, Gebretsadik T, Shintani A, Stein CM (2008). Novel cardiovascular risk factors in premature coronary atherosclerosis associated with systemic lupus erythematosus. J Rheumatol.

[14] Hua X, Su J, Svenungsson E, Hurt-Camejo E, Jensen-Urstad K, Angelin B, Båvenholm P, Frostegård J (2009). Dyslipidaemia and lipoprotein pattern in systemic lupus erythematosus (SLE) and SLE-related cardiovascular disease. Scand J Rheumatol.

[15] Parra S, Vives G, Ferré R, González M, Guardiola M, Ribalta J, Castro A (2012). Complement system and small HDL particles are associated with subclinical atherosclerosis in SLE patients. Atherosclerosis.

[16] Navab M, Hama SY, Hough GP, Subbanagounder G, Reddy ST, Fogelman AM (2001). A cell-free assay for detecting HDL that is dysfunctional in preventing the formation of or inactivating oxidized phospholipids. J Lipid Res.

[17] McMahon M, Grossman J, FitzGerald J, Dahlin-Lee E, Wallace DJ, Thong BY, Badsha H, Kalunian K, Charles C, Navab M (2006). Proinflammatory high-density lipoprotein as a biomarker for atherosclerosis in patients with systemic lupus erythematosus and rheumatoid arthritis. Arthritis Rheum.

[18] McMahon M, Grossman J, Skaggs B, Fitzgerald J, Sahakian L, Ragavendra N, Charles-Schoeman C, Watson K, Wong WK, Volkmann E (2009). Dysfunctional proinflammatory high-density lipoproteins confer increased risk of atherosclerosis in women with systemic lupus erythematosus. Arthritis Rheum.

[19] Gaál K, Lőrincz H, Seres I, Harangi M, Oláh AV, Paragh G (2013). Characterization of a novel high-density lipoprotein antioxidant capacity assay and its application to high-density lipoprotein fractions. Clin Biochem.

[20] Hochberg MC (1997). Updating the American College of Rheumatology revised criteria for the classification of systemic lupus erythematosus. Arthritis Rheum.

[21] Ansell BJ, Navab M, Hama S, Kamranpour N, Fonarow G, Hough G, Rahmani S, Mottahedeh R, Dave R, Reddy ST, Fogelman AM (2003). Inflammatory/antiinflammatory properties of high-density lipoprotein distinguish patients from control subjects better than high-density lipoprotein cholesterol levels and are favorably affected by simvastatin treatment. Circulation.

[22] Kalantar-Zadeh K, Kopple JD, Kamranpour N, Fogelman AM, Navab M (2007). HDL-inflammatory index correlates with poor outcome in hemodialysis patients. Kidney Int.

[23] Ergüder IB, Ergüder T, Ozkan C, Bozkurt N, Soylu K, Devrim E, Durak I (2006). Short-term effects of smoking cessation on blood antioxidant parameters and paraoxonase activity in healthy asymptomatic long-term cigarette smokers. Inhal Toxicol.

[24] Solak ZA, Kabaroğlu C, Cok G, Parildar Z, Bayindir U, Ozmen D, Bayindir O (2005). Effect of different levels of cigarette smoking on lipid peroxidation, glutathione enzymes and paraoxonase 1 activity in healthy people. Clin Exp Med.

[25] Tan EM, Cohen AS, Fries JF, Masi AT, McShane DJ, Rothfield NF, Schaller JG, Talal N, Winchester RJ (1982). The 1982 revised criteria for the classification of systemic lupus erythematosus. Arthritis Rheum.

[26] Ujhelyi L, Balla J, Muszbek L, Kakuk G, Belcher J, Jacob HS, Vercellotti GM, Balla G (1998). A microassay to assess the oxidative resistance of low-density lipoproteins. Clin Chem.

[27] McCall MR, Tang JY, Bielicki JK, Forte TM (1995). Inhibition of lecithin-cholesterol acyltransferase and modification of HDL apolipoproteins by aldehydes. Arterioscler Thromb Vasc Biol.

[28] Voros S, Joshi P, Qian Z, Rinehart S, Vazquez-Figueroa JG, Anderson H, Elashoff M, Murrieta L, Karmpaliotis D, Kalynych A (2013). Apoprotein B, small-dense LDL and impaired HDL remodeling is associated with larger plaque burden and more noncalcified plaque as assessed by coronary CT angiography and intravascular ultrasound with radiofrequency backscatter: results from the ATLANTA I study. J Am Heart Assoc.

[29] Kwankaew J, Leelawattana R, Saignam A, Siripaitoon B, Uea-Areewongsa P, Juthong S (2015). Apolipoprotein B as an independent predictor of arterial stiffness in systemic lupus erythematosus patients. Int J Rheum Dis.

[30] Hiruta N, Uchida Y, Maezawa Y, Shimoyama E (2013). Molecular imaging of apolipoprotein B-100 in human coronary plaques by color fluorescent angioscopy and microscopy. Int Heart J.

